# Supplementary material for: Understanding public opinion to the introduction of minimum unit pricing in Scotland: a qualitative study using Twitter
Source: BMJ Open. 2019 Jun 14;9(6):e029690. doi: 10.1136/bmjopen-2019-029690 (PMC6576115; doi:10.1136/bmjopen-2019-029690)
Supplement: Author's manuscript [file bmjopen-2019-029690.draft_revisions.pdf]

BMJ Open is committed to open peer review. As part of this commitment we make the peer review history of every article we publish publicly available.

When an article is published we post the peer reviewers' comments and the authors' responses online. We also post the versions of the paper that were used during peer review. These are the versions that the peer review comments apply to.

The versions of the paper that follow are the versions that were submitted during the peer review process. They are not the versions of record or the final published versions. They should not be cited or distributed as the published version of this manuscript.

BMJ Open is an open access journal and the full, final, typeset and author-corrected version of record of the manuscript is available on our site with no access controls, subscription charges or pay-per-view fees (http://bmjopen.bmj.com).

If you have any questions on BMJ Open's open peer review process please email info.bmjopen@bmj.com

BMJ Open

Understanding public opinion to the introduction of Minimum Unit Pricing in Scotland: a qualitative study using Twitter

Journal:	*BMJ Open*
Manuscript ID	bmjopen-2019-029690
Article Type:	Research
Date Submitted by the Author:	05-Feb-2019

Complete List of Authors:	Astill Wright, Laurence; University of York, Department of Health Sciences<br>Golder, Su; University of York,<br>Balkham, Adam; University of York, Department of Health Sciences<br>McCambridge, J; University of York, Department of Health Sciences

Keywords:	Alcohol, Scotland, Pubic Policy, Public Opinion, Social Media, Internet

SCHOLARONE™
Manuscripts

Paper type:

Original paper

Manuscript title:

Understanding public opinion to the introduction of Minimum Unit Pricing in Scotland: a qualitative study using Twitter

Author names and affiliations:
Laurence Astill Wright[1], Su Golder[1], Adam Balkham[1] and Jim McCambridge[1]

[1]Department of Health Sciences, University of York, UK

Correspondence to:
Laurence Astill Wright
Department of Health Sciences
University of York
Heslington
York
YO10 5DD
Tel: 01904321344
Fax: 01904 321383
laurencewright@doctors.org.uk

Keywords
Ethanol
Scotland
Public Policy
Public Opinion
Social Media
Internet

Word count:

3117

Abstract

Background and Aims
On 1st May 2018 Minimum Unit Pricing (MUP) of alcohol was introduced in Scotland. This study aimed to assess responses to the policy implementation in comments made on Twitter.

Methods
All tweets relating to MUP were captured during the two weeks after the introduction of the policy. These tweets were assessed using a mixture of human and machine coding for relevance, sentiment and source. A thematic analysis was conducted.

Results
74,639 tweets were collected over 14 days. Study findings demonstrate that opinion on the introduction of MUP in Scotland is divided, as far as is discernible on Twitter, with a slightly higher proportion of positive posts, particularly in Scotland itself. Furthermore, 55% of positive tweets/retweets were originally made by health or alcohol policy-related individuals or organisations. Thematic analysis of tweets showed some evidence of misunderstanding around policy issues.

Conclusions

For peer review only - http://bmjopen.bmj.com/site/about/guidelines.xhtml

It is possible to appreciate the divided nature of public opinion on the introduction of MUP in Scotland using Twitter, the nature of the sentiment around it, and key actors involved, and it will be possible to later study how this changes when the policy becomes more established.

Declaration of Interests
We declare no competing interests.

Strengths and Limitations

- This is the largest social media analysis conducted on alcohol policy with analysis of 53,574 relevant tweets and the first to use a mixture of human and machine classification
- Classification was not perfect but agreement between coders was very good
- Twitter is not representative of the general population

For peer review only

For peer review only - http://bmjopen.bmj.com/site/about/guidelines.xhtml

Background

Over the last 40 years the relative price of alcohol has decreased significantly in many countries throughout the world. Alcohol has never been as widely available and affordable as it currently is and this is primarily due to taxation falling behind increased earnings and inflation.[1] To combat the 3.3 million deaths worldwide each year and 5.1% of the global burden of disease, the World Health Organisation (WHO) recommends appropriate taxation and pricing policy in order to increase the cost of alcohol as part of an overall public health strategy to reduce harmful drinking.[2,3]

Increasing alcohol prices consistently reduces consumption[4] and a minimum unit price (MUP) of 50p in Scotland is forecasted to decrease the consumption of harmful drinkers by 7%, hazardous drinkers by 2.5% and moderate drinkers by 1.2%.[5] Changes in taxation alone would require a 70% increase to cause similar reductions of 7% in consumption by harmful drinkers.[5] In comparison, minimum unit pricing (MUP) specifically targets the cheapest drinks favoured by the heaviest drinkers.[4]

In the second half of the twentieth century Scotland has struggled with the increasing health, social and economic consequences of greater alcohol consumption more so than the rest of the United Kingdom (UK).[6] Average weekly unit consumption and rates of chronic liver disease and cirrhosis are higher than in England and Wales.[6] There are significant economic costs in healthcare provision, crime, and lost productivity.[8] It is predicted that MUP will reduce the number of deaths due to alcohol by 60, hospital admissions by 1300 and crimes by 3500 in the first year alone.[5]

After a series of legal challenges and national debate lasting five years, MUP was approved by the UK Supreme Court and was introduced to Scotland on 1 May 2018.[9] It has been demonstrated that alcohol industry submissions made to a Scottish government consultation in 2008 misrepresented the peer reviewed literature surrounding alcohol health policy. [31] The arguments made against MUP during this consultation, such as the concern of a new black market alcohol industry, were reiterated by Scottish and UK newspapers in 2011 and 2012. Some newspapers argued that MUP would be ineffective and it would punish responsible drinkers and the poor, while those that advocated for MUP argued that it would reduce health and social harms. [7]

Public perception appears to be changing over time on MUP with the 2015 British Social Attitudes survey suggesting that 52% of British adults support MUP, 25% are against it and 22% are unsure,[10] compared to a British 2011 YouGov survey which suggested 47% and 44% for and against it respectively, while 9% were unsure.[11] A 2011 focus group study had suggested that British participants held largely negative attitudes towards MUP due to ‘a misunderstanding of the minimum price per unit policy itself’ and ‘the failure to recognise the significance of small incremental reductions in alcohol consumption’.[12] A further focus group study identified beliefs consistently associated with negative attitudes of pricing policy;[13] pricing policies will make no difference to behaviour, the government considers the national economy to be more important than the health of the general public and that government cannot be trusted.[13]

When comparing the popularity of alcohol policy approaches in a discrete choice experiment, Pechey et al.[14] demonstrate that MUP is less favoured than both regulating alcohol marketing and decreasing the number of alcohol sales outlets when consumption, health and social outcomes are not considered. However, Pechy et al.[14] do show that the popularity of MUP increases from 43% to 63% when considering its significant effects in reducing consumption and social harms. In the UK there are no robust relationships between socioeconomic status and support for alcohol policy options.[15] In other countries it has been found that heavier drinkers (whose drinking is most damaging to themselves and others) are less supportive of alcohol policy change.[16,17] Pechey et al.[14] suggest that policy makers should focus on the beneficial outcomes when advocating for MUP to increase public support.

Few studies have used online social media to try to ascertain public attitudes towards change in alcohol policy. Twitter is a social networking website where users can broadcast their opinions to a public audience. As of late 2017, Twitter had 330 million active monthly users[18] and has great potential as a resource for quantitative and qualitative analysis of public opinion. Stautz et al.[19] analysed the reaction to the updated UK alcohol guidelines in 2017, identifying that the majority of tweets were unsupportive of the adjustments,

For peer review only - http://bmjopen.bmj.com/site/about/guidelines.xhtml

which reduced the advised limits for low risk drinking downwards for men, and that the community as a whole was largely opposed to alcohol policy measures. No other studies have attempted to assess public reaction to alcohol-related policy changes using Twitter, although studies have assessed the perception of cannabis use,[20] electronic cigarettes[21] and electronic cigarette marketing.[22]

This study used Twitter posts to quantify sentiment expressed online during the introduction of MUP, conducts a thematic analysis of these perceptions, and analyses which Twitter users are associated with which particular sentiments. Our specific research questions were as follows: 1. What are the proportions of positive, negative and neutral tweets? 2. What themes are commonly expressed?3. Which Twitter users are expressing which themes? 4. Do the results mirror population survey data and other qualitative research surrounding MUP?

Methods

Data were collected from Twitter using the Gnip PowerTrack firehose provided by Discovertext (a text analytics software - https://discovertext.com). Discovertext, which has been used in previous Twitter research,[22] was also used to archive and machine-code tweets. Data collection started on 29 April 2018, with the introduction of MUP on 1 May 2018. Due to the large volume of tweets collected, data collection was stopped at 14 days ending on 12th May 2018. Research methods were in accordance with Rivers' and Lewis'[23] recommendations for the ethical use of Twitter data.

Search terms were trialled using Twitter's free search Application Programming Interface (API - https://twitter.com/search-advanced). Terms that produced more than one search result relating to MUP on the first page of search results were included. Different terms and spellings were trialled, and hashtags that were repeatedly mentioned in tweets were included. Slang terms for alcohol were identified using online thesauruses (e.g www.urbandictionary.com) and promising search terms included. Only English language tweets were included. The Twitter firehose was used to collect all publicly available tweets corresponding to the relevant search terms.

The final search strategy was:

((minimum unit price) OR (minimum unit pricing) OR (minimum pricing) OR (minimum price) OR (minimum alcohol price) OR (minimum alcohol pricing) OR (minimum booze price) OR (minimum booze pricing) OR (min booze price) OR (min booze pricing) OR (min unit price) OR (min unit pricing) OR (min alcohol price) OR (min alcohol pricing) OR (MUP) OR (50p unit) OR (Scotland alcohol) OR (Scotland booze) OR (Scotland bevy) OR (Scotland min price) OR (Scotland min pricing) OR (alcohol unit) OR (minimum price per unit) OR (cheap booze Scotland) OR (minimum cost alcohol) OR (min cost alcohol) OR #minimumunitpricing OR #mupsaveslives OR #MUP) lang:en

Tweets were initially coded as relevant or irrelevant by a single human coder. The human coder's endeavours were used to train Discovertext's machine classifier using a Naive Bayes algorithm. This allows machine coding of remaining tweets and was then applied to the full selection of tweets. While the algorithm was excellent at excluding irrelevant tweets, sometimes these irrelevant tweets were incorrectly classified as relevant. This was an iterative process and so the machine classifier was retrained until it reached an acceptable degree of accuracy. The agreement between machine classifier and human coder (LAW) was calculated using a kappa score on an overlapping selection of 100 tweets. The human coder's work was validated against a second human coder (AB) on an overlapping selection of 500 tweets. Irrelevant tweets were then discarded.

Once relevant tweets were separated from irrelevant, a similar process was used to classify tweets according to sentiment. A single human coder classified relevant tweets into positive, negative and neutral. The coding of the primary coder was validated against the coding of the second human coder using a kappa score on an overlapping sample of 200 tweets. Series of 200 tweets were double classified until kappa scores greater than 0.7 were achieved and this was used to train a new custom machine classifier that was applied to all the relevant tweets. Inaccuracies in machine coding were refined by human coding of key tweets to retrain the

For peer review only - http://bmjopen.bmj.com/site/about/guidelines.xhtml

algorithm. Again, machine coding was validated using a kappa score on an overlapping sample of 100 tweets.

Once relevant tweets were separated into positive, negative and neutral, a random sample of 500 tweets was taken from each of the three subgroups using DiscoverText's random sampling tool. These 1500 tweets were analysed and single coded to assess the predominant themes. Prior to assessment we reviewed previous media arguments for and against MUP,[7] and various public surveys[24] to establish the range of anticipated themes (4 positive, 8 negative). This was an iterative process and when a theme was not congruent with the anticipated themes, it was considered a newly emerging theme and this was added. 2 new themes emerged through this process, 1 positive and 1 negative.

New themes, in addition to those already identified, emerged only in the initial stages of analysis and no new themes emerged in the later stages of analysis (the final 150 tweets of each 500 tweet sample). Thus it was determined that sufficient saturation had been reached, and no additional tweets needed to be examined. The popularity of each theme was also assessed in each random sample.

These three random samples of 500 tweets were also analysed to determine the source of the tweets. A single human coder examined each author's Twitter page. For each tweet/retweet, the username, full name, associated biography and the associated results from an internet search engine (https://www.google.com) were examined to determine the user's background. The same process was used to determine if the source self identified as Scottish or lived in Scotland or not (only 1.6% of Twitter users have their geolocation activated and so inferences must be made from their profile).[25] For example, Twitter profiles contain a space for a user to write their location and if this was a Scottish place it was assumed that the user was Scottish. Some users did not write a location but had explicit references to the place they lived in their tweets. A chi-squared test was used to determine if there was statistical significance (p-value <0.05) between location and sentiment.

Results

74,639 tweets were collected over 14 days. 62,879 of these tweets were manually coded as either 'relevant' to MUP or 'not relevant' by the same coder (LAW). The Naive Bayes algorithm subsequently coded the remaining 11,760 tweets. 500 tweets were coded by both the primary coder and a second coder (AB). Of these 500 tweets, there was a 97% agreement with a kappa score of 0.95. This indicates an excellent level of agreement. In order to validate the coding of the algorithm, 100 tweets were coded by both the primary coder (LAW) and the algorithm. For these 100 tweets there was a 97% agreement with a kappa score of 0.94, providing further reassurance about reliability on relevance.

53,574 (72%) tweets were classified as relevant, while 21,065 (28%) were classified as 'not relevant'. The irrelevant tweets made no reference to the MUP of alcohol in any context. These 53,574 relevant tweets were subsequently classified according to sentiment. 57,801 tweets were manually coded. 18,741 were coded as positive (35%), 14,866 as negative (28%), 17,302 as neutral (32%), and 2665 as not relevant (5%). In the 200 tweets coded by both the primary and secondary coder there was a kappa score of 0.75. The kappa scores were: positive - 0.79, negative - 0.74, neutral - 0.76, not relevant - 0.73. This shows good agreement for sentiment tweets. For 100 tweets coded by both the primary coder (LAW) and the algorithm there was a 96% agreement with a kappa score of 0.94.

From each sentiment (positive, negative and neutral) 500 randomly selected tweets were analysed for predominant themes. These were elaborated through the process of thematically coding each tweet and new themes were added as they occurred until saturation was reached. Twitter based thematic analysis is difficult to automate using machine algorithms due to the abbreviations, emoticons and sarcasm[26] and we relied exclusively on human coding. The findings are presented in Table 1. Perceived ability to reduce health harms was the most prominent theme in the positive tweets, skepticism about effects on problem drinkers was the most prominent in the negative tweets, and factual information were the most prominent theme in the neutral tweets. In each of these sentiment categories a small proportion of tweets (ranging from 1.6-5.2%) were found to be misclassified. Some were irrelevant but falsely classified as relevant, while some were of another sentiment. It is impossible to say whether it was human or machine coding which produced this error.

Table 1: Results of thematic analysis of positive, negative and neutral tweets with paraphrased examples

For peer review only - http://bmjopen.bmj.com/site/about/guidelines.xhtml

Theme of positive tweets	n	%	Paraphrased example
Reduces health harms	352	70.4	Minimum Unit Pricing will decrease hospital admissions and save lives #mupsaveslives
Reduces social harms	13	2.6	This will greatly reduce alcohol-fuelled violence and other countries must follow
Effectively targets the cheapest, strongest alcohol	36	7.2	Strong cider sold at pocket money prices is hugely damaging
Scotland has an alcohol problem and something must be done	26	5.2	This country has an awful relationship with drink - let's try MUP
MUP is an evidence-based policy	5	1.0	The evidence backs MUP, which has been approved by the courts and will be extensively evaluated with a sunset clause
Nil reason given	60	12.0	Excellent work from the SNP!
Incorrectly classified as positive	8	1.6	
Total	500	100	
Theme of negative tweets	*n*	*%*	*Paraphrased example*
Alcoholics will not decrease their alcohol intake	138	27.6	Alcoholics will not buy less but their children will go without so they can get it
Increase in illicit alcohol production and/or encourage cross-border trading	71	14.2	Hoards will rush over the border to stock up on frosty jacks - who would've thought we'd have a booze cruise in 2018
Anti-libertarian	54	10.8	First the sugar tax and now this - the nanny state won't stop
A tax on the poor	52	10.4	Another example of a classist poor-bashing policy
Increase in drug use and/or petty crime	23	4.6	Neds will rob grannies for booze money and the jakeys will turn to drugs instead
Punishes responsible drinkers	17	3.4	A few people can't drink responsibly and now everyone else has to pay the price?
Increases retailer profits	6	1.2	All this will do is line the pockets of billionaires - the supermarkets can't believe their luck
Harms businesses	2	0.4	How many jobs will be lost from this?
Alcohol consumption is a cultural problem	3	0.6	Other countries with cheap alcohol don't have the same problems - the problem isn't to do with the price
Nil reason given	108	21.6	This new alcohol law is embarrassing bs #SNPfail
Incorrectly classified as negative	26	5.2	
Total	500	100	
Theme of neutral tweets	*n*	*%*	*Paraphrased example*
Factual	301	60	Scotland introduces new alcohol law
Humour	102	20	Great that Scotland are adopting the alcohol pricing design they have trialled for so long at the Edinburgh fringe
Balanced/Unclear Sentiment	82	16	On the one hand it could reduce overconsumption of alcohol, but on the other it could encourage a black market
Incorrectly classified as neutral	15	3	

For peer review only - http://bmjopen.bmj.com/site/about/guidelines.xhtml

The random samples of 500 tweets divided by sentiment were next classified according to the background of the Twitter user who posted the tweet. In the case of retweets this was the original tweeter. It was not possible to determine who retweets were made by. The users were divided up into the groups as in Table 2. Miscellaneous users were those accounts who did not fall into any of the other groups and largely consisted of private companies and spam accounts.

Table 2: Source analysis of tweets and retweets from positive, negative and neutral sub-groups

		Original source of tweet or retweet							
		Member of public	*Health/alcohol policy organisation/individual*	*Media/news organisation/individual*	*Alcohol industry-related organisation/individual*	*Celebrity/ public figure*	*Miscellaneous*	*Incorrectly classified as positive/negative/neutral*	*Total*
Positive	*n*	85	275	91	1	17	25	6	500
	%	17	55	18.2	0.2	3.4	5.0	1.2	100
Negative	*n*	287	53	56	5	2	81	16	500
	%	57.4	10.6	11.2	1.0	0.4	16.2	3.2	100
Neutral	*n*	189	45	109	18	10	120	9	500
	%	37.8	9.0	21.8	3.6	2.0	24	1.8	100

When discerning if the source self identified as Scottish or lived in Scotland or not, we used a chi-square test and found that positive tweets were more often to be from Scottish Twitter accounts (see Table 3) (p-value 0.002). Negative tweets were just as likely to be from not obviously Scottish twitter accounts (p-value 0.066) and neutral tweets were less likely to be from Scottish twitter accounts (p-value 0.001).

Table 3: Source nationality analysis of tweets and retweets from positive, negative and neutral subgroups

		Source nationality				
		Likely Scottish	*Not obviously Scottish*	*Unable to view profile*	*Not correctly assigned the right sentiment*	*Total*
Positive	*n*	293	187	14	6	500
	%	58.6	37.4	2.8	1.2	100
Negative	*n*	218	204	65	13	500
	%	43.6	40.8	13	2.6	100
Neutral	*n*	219	259	13	9	500
	%	43.8	51.8	2.6	1.8	100

For peer review only - http://bmjopen.bmj.com/site/about/guidelines.xhtml

Discussion

Study findings demonstrate that public opinion on the introduction of MUP in Scotland was divided, with a slightly higher proportion of positive posts than negative or neutral, particularly in Scotland itself. These findings mirror previous survey data that suggest a growing proportion of the British public favour MUP than are against it.[10]

Public opinion alone does not dictate alcohol policy and there is often significant industry and political will to resist change. There do, however, remain complex interactions between public opinion and shifts in alcohol policy. Österberg and colleagues[27] demonstrate that a decrease in alcohol excise duty in Finland in 2004 and a subsequent rise in alcohol related harm led to an increase in support for alcohol policies to counteract these trends. In Ireland high levels of alcohol consumption and a doubling of alcohol related street violence over seven years led to public discussion which culminated in increased alcohol taxation, via increased support for alcohol policies.[28] There is some suggestion in this study and the literature on which it draws that Scotland has followed a similar pattern to Finland and Ireland where it appears that an increase in alcohol harms has prompted public discussion putting alcohol policy change on policy agendas. A more nuanced historical study would be needed to investigate how far this is true, and the roles of political actors in relation to public opinion.[29,30]

In 1984 John Kingdon proposed that shifts in public policy require the overlapping of three different factors - the public acknowledgement of a problem, a clear solution to a problem, and also the political will to address the issue. [31] In relation to MUP there was first a public discussion of the harms of alcohol consumption. Researchers and public health experts subsequently paid more attention to restrictive alcohol policies as a solution to this problem, and then the SNP showed the political will to address these alcohol harms. These three factors may have overlapped to create a unique 'window of opportunity' to introduce MUP.

Only one other study has examined social media responses (analysing 3061 tweets) to alcohol policy-related developments.[19] The present study is thus the largest conducted on alcohol policy with analysis of 53,574 relevant tweets and the first to use a mixture of human and machine classification. Stautz et al.[19] showed a predominantly negative reaction to updated alcohol guidelines (27.4% negative vs 6.8% positive). There are several interpretations of the large difference in sentiment between the updated alcohol guidelines and the introduction of MUP. While it is possible that public support for MUP is far greater, it is also possible that because Stautz et al.[19] only followed one hashtag (while we followed 29 different synonyms accounting for different terminology and spellings), this procedure yielded a less representative sample. In our study the most popular hashtag relating to MUP was only used in 3.8% of relevant tweets and so we would not recommend searching based on hashtags alone when conducting future alcohol policy-related research.

Thematic analysis of positive tweets showed less variation in arguments supporting MUP than against it. 70.4% of positive tweets focused on the health benefits of MUP and a minority focused on other views. This reflects the introduction of MUP for primarily public health reasons. Furthermore, health/alcohol policy organisations/individuals tweets or retweets were the original sources of the majority of positive tweets surrounding MUP. This suggested a coordinated response by health care professionals and public health organisations focusing on a single message - that MUP reduces alcohol-related health problems. These findings highlight the important implications for advocacy groups of investing in social media to influence public opinion.

Additionally, our study found that 24.9% of the 1500 randomly selected sentiment tweets were made by health/alcohol policy-related Twitter accounts, while Stautz et al.[19] demonstrated 12.4% of tweets relating to the updated UK alcohol guidelines were made by health related individuals/organisations. The responses of health advocacy groups to MUP appears to be more effective than that to alcohol guidelines, in part because of the ongoing failure to implement UK public information campaigns on the new guidelines, and the remarkable refusal of alcohol producers to carry the revised guidelines on alcohol packaging. As Pechey et al.[14] recommend, Scottish policy-makers have given prominence to the expected outcomes of MUP.

For peer review only - http://bmjopen.bmj.com/site/about/guidelines.xhtml

Thus we suggest that when engaging with social media, public health advocacy groups should be aware of the potential reach of their messages and use this as an opportunity to advance public understanding of the issues and support for policy measures underpinned by evidence, focusing on the anticipated outcomes specifically.

Many of the negative themes expressed were similar to alcohol industry framings of the issues from earlier on in the public debate.[32,33] Following on from the industry's attempts to obstruct the implementation of MUP through legal processes, the alcohol actors we identified through Twitter continued to propagate the negative framing of MUP in an attempt to marginalise those arguments based on peer-reviewed literature. Yet, by the time of implementation of the policy, it is striking how little such activity there was by industry actors.

The similarity we have demonstrated in findings between Twitter-based research to gauge public perceptions and general population surveys may provide some support for social media-based methods as adjuncts to survey based opinion polling. We showed that 35% of tweets were positive, 28% were negative and 32% were neutral. Similarly the 2011 YouGov survey suggested that 47% supported MUP, while 44% opposed it and 9% were unsure. [11] Likewise the 2015 British Social Attitudes survey suggests that 52% of British adults support MUP, while 25% are against it and 22% are unsure. [10] Gauging public opinion via social media has numerous practical advantages over polling, though validation methods remain to be developed. There will probably be lower costs given that the data are already in the public domain, and machine algorithms can be used to code items with high inter-rater reliability with human coders. Social media research does, however, bring with it new ethical challenges that must be considered by future researchers.[34]

The limitations of our work include a degree of uncertainty about the inferences about public opinion that can be made from this data. Twitter users are unlikely to be representative of the general population, as they are more likely to be urban dwelling, male, and have higher educational achievement.[35,36] Twitter users tend to hold more extreme views[37] and surround themselves with those who hold similar opinions (in what is know as echo-chambers).[38] This makes inferences in relation to previous polling data questionable. Furthermore, classification was not perfect and 3.3% of tweets were included in the wrong sentiment group in our random sample of 1500 tweets. It is also possible our results were subject to confounding, for example, political affiliation. Many accounts provided limited biographical information and so this was not measured or adjusted for. Nonetheless, it is possible to appreciate the divided nature of public opinion on the introduction of MUP, the nature of the sentiment around it, and key actors involved, and it will be possible to later study how this changes when the policy becomes more established.

Ethical Approval

The University of York Health Sciences Research Governance Committee was consulted who recommended that the study did not require full ethical approval as the Twitter data used were already in the public domain.

Contributors

LAW, SG and JM were responsible for the original study design. LAW was responsible for primary data coding, analysis and for initial drafting of this report. AB was the second coder. LAW, SG, AB and JM were responsible for subsequent interpretation, editing and rewriting for the report.

Funding

This work was supported by a Wellcome Trust Investigator Award in Humanities and Social Science (200321/Z/15/Z) to JM.

Declaration of Interests

We declare no competing interests.

References

1. Babor T. Caetano R. 2010. Alcohol: No Ordinary Commodity. 2nd edition. New York: Oxford University Press. ISBN: 0199551146.

For peer review only - http://bmjopen.bmj.com/site/about/guidelines.xhtml

2. Monteiro M. 2011. The Road to a World Health Organization Global Strategy for Reducing the Harmful Use of Alcohol. Alcohol Res Health. 34(2). PMID: 22330226.

3. World Health Organization. 2010. Global Strategy to Reduce Harmful Use of Alcohol [Online]. Available at: http://www.who.int/substance_abuse/activities/gsrhua/en/. Accessed 16/08/2018 (Archived by WebCite® at http://www.webcitation.org/71mmbuaiB).

4. Sharma A. Sinha K. Vandenberg, B. 2017. Pricing as a means of controlling alcohol consumption. Br Med Bull. 123(1). PMID: 28910991.

5. Angus C. Holmes J. Pryce R. Meier P. Brennan A. 2016. Model-based appraisal of the comparative impact of Minimum Unit Pricing and taxation policies in Scotland. An adaptation of the Sheffield Alcohol Policy Model version 3 [Online]. Available at: https://www.sheffield.ac.uk/polopoly_fs/1.565373!/file/Scotland_report_2016.pdf. Accessed: 06/05/18 (Archived by WebCite® at http://www.webcitation.org/71mlo3iju).

6. Leon D. McCambridge J. (2006). Liver cirrhosis mortality rates in Britain from 1950 to 2002: an analysis of routine data. Lancet. 367(9504). PMID: 16399153.

7. Hilton S. Wood K. Patterson C. Katikireddi, S. 2014. Implications for alcohol minimum unit pricing advocacy: What can we learn for public health from UK newsprint coverage of key claim-makers in the policy debate? Soc Sci Med. 102(100). PMID: 24565153.

8. Scottish Government. 2008. Changing Scotland's relationship with alcohol: a discussion paper on our strategic approach [Online]. Available at: http://www.gov.scot/Resource/Doc/227785/0061677.pdf. Accessed: 06/05/18 (Archived by WebCite® at http://www.webcitation.org/71mmCjRMw).

9. Meier P. Brennan A. Angus C. Holmes J. 2017. Minimum unit pricing for alcohol clears final legal hurdle in Scotland. BMJ. 359. PMID: 29162561.

10. NatCen Social Research. 2015. Attitudes to alcohol. Findings from the 2015 British Social Attitudes survey [Online]. Available at: http://bsa.natcen.ac.uk/media/39126/bsa-attitudes-to-alcohol-final.pdf. Accessed: 09/05/2018 (Archived by WebCite® at http://www.webcitation.org/71mm05C1f).

11. YouGov. 2011. 'Pocket money' prices [Online]. Available at: https://yougov.co.uk/news/2011/01/25/pocket-money-prices-alcohol/. Accessed: 07/05/2018 (Archived by WebCite® at http://www.webcitation.org/71mmQgn15).

12. Hagger M. Lonsdale A. Baggott R. Penny G. Bowen M. 2011. The Cost of Alcohol: The Advocacy for a Minimum Price per Unit in the UK [Online]. Available at: http://alcoholresearchuk.org/downloads/finalReports/FinalReport_0082. Accessed 07/05/18 (Archived by WebCite® at http://www.webcitation.org/71mltCAb8).

13. Somerville C. Marteau T. Kinmonth A. Cohn S. 2015. Public attitudes towards pricing policies to change health-related behaviours: a UK focus group study. Eur J Public Health. 25(6). PMID: 25983329.

14. Pechey R. Burge P. Mentzakis E. Suhrcke M. Marteau M. 2014. Public acceptability of population-level interventions to reduce alcohol consumption: a discrete choice experiment. Soc Sci Med. 113. PMID: 24858928.

15. Erskine S. Maheswaran R. Pearson T. Gleeson D. 2010. Socioeconomic deprivation, urban-rural location and alcohol-related mortality in England and Wales. BMC Public Health. 10(99). PMID: 20184763.

For peer review only - http://bmjopen.bmj.com/site/about/guidelines.xhtml

For peer review only

16. Giesbrecht N. Lalomiteanu A. Anglin L. Adlaf E. 2007. Alcohol marketing and retailing: Public opinion and recent policy developments in Canada. Journal of Substance Use. 6. doi:10.1080/14659890701262189.

17. Giesbrecht N. and Livingston M. 2014. Public perceptions and alcohol policies: Six case studies that examine trends and interactions. Drug Alcohol Rev. 33(3). doi:10.1111/dar.12139.

18. Statista. 2018. Number of monthly active Twitter users worldwide from 1st quarter 2010 to 4th quarter 2017 (in millions) [Online]. Available at: https://www.statista.com/statistics/282087/number-of-monthly-active-twitter-users/. Accessed: 08/05/2018 (Archived by WebCite® at http://www.webcitation.org/71mmJR0Bd).

19. Stautz K. Bignardi G. Hollands G. Marteau T. et al. 2017. Reactions on Twitter to updated alcohol guidelines in the UK: a content analysis. BMJ Open 7(2). doi:10.1136/bmjopen-2016-015493.

20. Thompson L. Rivara F. Whitehill J. 2013. Prevalence of Marijuana-Related Traffic on Twitter, 2012–2013: A Content Analysis. Cyberpsychol Behav Soc Netw. 18(6). PMID: 26075917.

21. Cole-Lewis H. Pugatch J. Sanders A. Varghese A. Posada S. Yun, C. Schwarz M. Auguston E. 2015. Social Listening: A Content Analysis of E-Cigarette Discussions on Twitter. J Med Internet Res. 17(10). PMID: 26508089.

22. Huang J. Kornfield R. Szczypka G. Emery S. 2014. A cross-sectional examination of marketing of electronic cigarettes on Twitter. Tob Control. 23(3). PMID: 24935894.

23. Rivers C. Lewis B. 2014. Ethical research standards in a world of big data. F1000Research. 38(1-10). doi:10.12688/f1000research.3-38.v1.

24. Alcohol Policy UK. 2016. British Social Attitudes survey 2015: is support for minimum pricing growing? [Online]. Available at: http://www.alcoholpolicy.net/2016/09/social-survey-attitudes-2015-is-support-for-minimum-pricing-growing.html. Accessed 04/05/18 (Archived by WebCite® at http://www.webcitation.org/71mlXdIwf).

25. Leetaru K. Wang S. Cao G. Padmanabhan A. Shook E. 2013. Mapping the global Twitter heartbeat: The geography of Twitter. First Monday. 18(5). doi:10.5210/fm.v18i5.4366.

26. Thelwall M. Buckley K. Paltoglou G. 2012. Sentiment strength detection for the social Web. Journal of the American Society for Information Science and Technology. 63(1). doi:10.1002/asi.21662.

27. Österberg E. Lindeman M. Karlsson T. 2014. Changes in alcohol policies and public opinions in Finland 2003-2013. Drug Alcohol Rev. 33(3). PMID: 24628708.

28. Hope A. 2014. The ebb and flow of attitudes and policies on alcohol in Ireland 2002–2010. Drug Alcohol Rev. 33(3). PMID: 24628739.

29. Holden C. Hawkins B. 2014. 'Whisky gloss': The alcohol industry, devolution and policy communities in Scotland. Public Policy & Administration. 28. doi:10.1177/0952076712452290.

30. McCambridge J. Hawkins B. Holden C. 2014. Vested interests in addiction research and policy. The challenge corporate lobbying poses to reducing society's alcohol problems: insights from UK evidence on minimum unit pricing. Addiction. 109(2). PMID: 24261642.

31. Béland, D. Howlett, M. 2016. The Role and Impact of the Multiple-Streams Approach in Comparative Policy Analysis. Journal of Comparative Policy Analysis. 18(3).

For peer review only - http://bmjopen.bmj.com/site/about/guidelines.xhtml

32. McCambridge J. Hawkins B. Holden C. 2013. Industry use of evidence to influence alcohol policy: a case study of submissions to the 2008 Scottish government consultation. PLoS Med. 10(4). PMID: 23630458.

33. Hawkins B. McCambridge J. 2014. Industry Actors, Think Tanks, and Alcohol Policy in the United Kingdom. Am J Public Health. 104(8). PMID: 24922137.

34. Golder S. Ahmed S. Norman G. Booth A. 2017. Attitudes Toward the Ethics of Research Using Social Media: A Systematic Review. J Med Internet Res. 19(6). PMID: 28588006.

35. Mellon J. Prosser C. 2017. Twitter and Facebook are not representative of the general population: Political attitudes and demographics of British social media users. Research and Politics. 1(9). doi:10.1177/2053168017720008.

36. Mislove A. Lehmann S. Ahn Y. Onella J. Rosenquist J. 2011. Understanding the demographics of Twitter users. Proceedings of the Fifth International AAAI Conference on Weblogs and Social Media. AAAI Press. ISBN: 9781577355052.

37. Barberá P. Rivero G. 2014. Understanding the Political Representativeness of Twitter Users. Social Science Computer Review. 33(6). doi:10.1177/0894439314558836.

38. Garimella K. De Francisci Morales G. Gionis A. Mathioudakis M. 2018. Political Discourse on Social Media: Echo Chambers, Gatekeepers, and the Price of Bipartisanship. WWW 2018: The 2018 Web Conference. doi:10.1145/3178876.3186139.

For peer review only

For peer review only - http://bmjopen.bmj.com/site/about/guidelines.xhtml

Understanding public opinion to the introduction of Minimum Unit Pricing in Scotland: a qualitative study using Twitter

Journal:	*BMJ Open*
Manuscript ID	bmjopen-2019-029690.R1
Article Type:	Research
Date Submitted by the Author:	09-Apr-2019
Complete List of Authors:	Astill Wright, Laurence; University of York, Department of Health Sciences<br>Golder, Su; University of York, Department of Health Sciences<br>Balkham, Adam; University of York, Department of Health Sciences<br>McCambridge, J; University of York, Department of Health Sciences
**Primary Subject Heading**:	Public health
Secondary Subject Heading:	Addiction, Health policy, Mental health, Gastroenterology and hepatology
Keywords:	Alcohol, Scotland, Pubic Policy, Public Opinion, Social Media, Internet

SCHOLARONE™
Manuscripts

Paper type:

Original paper

Manuscript title:

Understanding public opinion to the introduction of Minimum Unit Pricing in Scotland: a qualitative study using Twitter

Author names and affiliations:
Laurence Astill Wright[1], Su Golder[1], Adam Balkham[1] and Jim McCambridge[1]

[1]Department of Health Sciences, University of York, UK

Correspondence to:
Laurence Astill Wright
Department of Health Sciences
University of York
Heslington
York
YO10 5DD
Tel: 01904321344
Fax: 01904 321383
laurencewright@doctors.org.uk

Keywords
Ethanol
Scotland
Public Policy
Public Opinion
Social Media
Internet

Word count:

3650

Abstract

Objectives
On 1st May 2018 Minimum Unit Pricing (MUP) of alcohol was introduced in Scotland. This study used Twitter posts to quantify sentiment expressed online during the introduction of MUP, conducted a thematic analysis of these perceptions, and analysed which Twitter users were associated with which particular sentiments.

Design and setting
This qualitative social media analysis captured all tweets relating to MUP during the two weeks after the introduction of the policy. These tweets were assessed using a mixture of human and machine coding for relevance, sentiment and source. A thematic analysis was conducted.

Participants
74,639 tweets were collected over 14 days. Of these 53,574 were relevant to MUP.

Results
Study findings demonstrate that opinion on the introduction of MUP in Scotland was somewhat divided, as far as is discernible on Twitter, with a slightly higher proportion of positive posts (35%) than negative posts

For peer review only
For peer review only - http://bmjopen.bmj.com/site/about/guidelines.xhtml

(28%), with positive sentiment stronger in Scotland itself. Furthermore, 55% of positive tweets/retweets were originally made by health or alcohol policy-related individuals or organisations. Thematic analysis of tweets showed some evidence of misunderstanding around policy issues.

Conclusions
It is possible to appreciate the divided nature of public opinion on the introduction of MUP in Scotland using Twitter, the nature of the sentiment around it, and the key actors involved. It will be possible to later study how this changes when the policy becomes more established.

Declaration of Interests
We declare no competing interests.

Strengths and Limitations
- This is the largest social media study conducted on alcohol policy with analysis of 53,574 relevant tweets
- This is the first alcohol policy study to use a mixture of human and machine classification
- Using the Twitter firehose and 29 synonyms in our search string maximised the number of Tweets collected
- Classification was not perfect but agreement between coders was very good
- Twitter is not representative of the general population

For peer review only

For peer review only - http://bmjopen.bmj.com/site/about/guidelines.xhtml

Background

Over the last 40 years the relative price of alcohol has decreased significantly in many countries throughout the world. Alcohol has never been as widely available and affordable as it currently is and this is primarily due to taxation falling behind increased earnings and inflation.[1] To combat the 3.3 million deaths worldwide each year and 5.1% of the global burden of disease, the World Health Organisation (WHO) recommends appropriate taxation and pricing policies in order to increase the cost of alcohol as part of an overall public health strategy to reduce harmful drinking.[2,3]

Increasing alcohol prices consistently reduces consumption[4] and a minimum unit price (MUP) of 50p in Scotland is forecast to decrease the consumption of harmful drinkers by 7%, hazardous drinkers by 2.5% and moderate drinkers by 1.2%.[5] Changes in taxation alone would require a 70% increase to cause a reduction of 7% in consumption by harmful drinkers.[5] In comparison, minimum unit pricing (MUP) specifically targets the cheapest drinks favoured by the heaviest drinkers.[4]

In the second half of the twentieth century Scotland has struggled with the increasing health, social and economic consequences of greater alcohol consumption more so than the rest of the United Kingdom (UK).[6] Average weekly unit consumption and rates of chronic liver disease and cirrhosis are higher than in England and Wales.[6] There are significant economic costs in healthcare provision, crime, and lost productivity.[7] It is predicted that MUP will reduce the number of deaths due to alcohol by 60, hospital admissions by 1300 and crimes by 3500 in the first year alone.[5]

After a series of legal challenges and national debate lasting approximately five years, MUP was approved by the UK Supreme Court and was introduced to Scotland on 1 May 2018.[8] It has been demonstrated that alcohol industry submissions made to a Scottish government consultation in 2008 misrepresented the peer reviewed literature surrounding alcohol policy. [9] The arguments made against MUP during this consultation, such as the concern of a new black market alcohol industry, were reiterated by Scottish and UK newspapers in 2011 and 2012. Some newspapers argued that MUP would be ineffective and it would punish responsible drinkers and the poor, while those that advocated for MUP argued that it would reduce health and social harms. [10]

Public perception appears to be changing over time on MUP with the 2015 British Social Attitudes survey suggesting that 52% of British adults support MUP, 25% are against it and 22% are unsure,[11] compared to a British 2011 YouGov survey which suggested 47% and 44% for and against it respectively, while 9% were unsure.[12] A 2011 focus group study had suggested that British participants held largely negative attitudes towards MUP due to 'a misunderstanding of the minimum price per unit policy itself' and 'the failure to recognise the significance of small incremental reductions in alcohol consumption'.[13] A further focus group study identified beliefs consistently associated with negative attitudes of pricing policy;[14] that pricing policies will make no difference to behaviour, the government considers the national economy to be more important than the health of the general public and that government cannot be trusted.[14]

When comparing the popularity of alcohol policy approaches in a discrete choice experiment, Pechey et al.[15] demonstrate that MUP is less favoured than both regulating alcohol marketing and decreasing the number of alcohol sales outlets when consumption, health and social outcomes are not considered. However, Pechy et al.[15] do show that the popularity of MUP increases from 43% to 63% when considering its significant effects in reducing consumption and social harms. In the UK there are no robust relationships between socioeconomic status and support for alcohol policy options**.**[16] In other countries it has been found that heavier drinkers (whose drinking is most damaging to themselves and others) are less supportive of alcohol policy change.[17,18] Pechey et al.[15] suggest that policy makers should focus on the beneficial outcomes when advocating for MUP to increase public support.

Few studies have used online social media to try to ascertain public attitudes towards change in alcohol policy. Twitter is a social networking website where users can broadcast their opinions to a public audience. As of late 2017, Twitter had 330 million active monthly users[19] and has great potential as a resource for quantitative and qualitative analysis of public opinion. Stautz et al.[20] analysed the reaction to the updated UK alcohol guidelines in 2017, identifying that the majority of tweets were unsupportive of the adjustments, which reduced the advised limits for low risk drinking downwards for men, and that the community as a

For peer review only - http://bmjopen.bmj.com/site/about/guidelines.xhtml

whole was largely opposed to alcohol policy measures. No other studies have attempted to assess public reaction to alcohol-related policy changes using Twitter, although studies have assessed the perception of cannabis use,[21] electronic cigarettes[22] and electronic cigarette marketing.[23]

This study used Twitter posts to quantify sentiment expressed online during the introduction of MUP, conducts a thematic analysis of these perceptions, and analyses which Twitter users are associated with which particular sentiments. Our specific research questions were as follows: 1. What are the proportions of positive, negative and neutral tweets? 2. What themes are commonly expressed? 3. Which Twitter users are expressing which themes? 4. Do the results mirror population survey data and other qualitative research surrounding MUP?

Methods

Data were collected from Twitter using the Gnip PowerTrack firehose provided by Discovertext (a text analytics software - https://discovertext.com). Discovertext, which has been used in previous Twitter research,[23] was also used to archive and machine-code tweets. Data collection started on 29 April 2018, with the introduction of MUP on 1 May 2018. Due to the large volume of tweets collected, data collection was stopped at 14 days ending on 12th May 2018. Research methods were in accordance with Rivers' and Lewis'[24] recommendations for the ethical use of Twitter data.

Search terms were trialled using Twitter's free search Application Programming Interface (API - https://twitter.com/search-advanced). Terms that produced more than one search result relating to MUP on the first page of search results were included. Different terms and spellings were trialled, and hashtags that were repeatedly mentioned in tweets were included. Slang terms for alcohol were identified using online thesauruses (e.g www.urbandictionary.com) and promising search terms included. Only English language tweets were included. The Twitter firehose was used to collect all publicly available tweets corresponding to the relevant search terms without the limitations of the Twitter API.

The final search strategy was:

((minimum unit price) OR (minimum unit pricing) OR (minimum pricing) OR (minimum price) OR (minimum alcohol price) OR (minimum alcohol pricing) OR (minimum booze price) OR (minimum booze pricing) OR (min booze price) OR (min booze pricing) OR (min unit price) OR (min unit pricing) OR (min alcohol price) OR (min alcohol pricing) OR (MUP) OR (50p unit) OR (Scotland alcohol) OR (Scotland booze) OR (Scotland bevy) OR (Scotland min price) OR (Scotland min pricing) OR (alcohol unit) OR (minimum price per unit) OR (cheap booze Scotland) OR (minimum cost alcohol) OR (min cost alcohol) OR #minimumunitpricing OR #mupsaveslives OR #MUP) lang:en

Tweets were initially coded as relevant or irrelevant by a single human coder. The human coder endeavours were used to train Discovertext's machine classifier using a Naive Bayes algorithm. This allows machine coding of remaining tweets and was then applied to the full selection of tweets. While the algorithm was excellent at excluding irrelevant tweets, sometimes these irrelevant tweets were incorrectly classified as relevant. This was an iterative process and so the machine classifier was retrained until it reached an acceptable degree of accuracy. The agreement between machine classifier and human coder (LAW) was calculated using a kappa score on an overlapping selection of 100 tweets. The human coder's work was validated against a second human coder (AB) on an overlapping selection of 500 tweets. Irrelevant tweets were then discarded.

Once relevant tweets were separated from irrelevant, a similar process was used to classify tweets according to sentiment. A single human coder classified relevant tweets into positive, negative and neutral. The coding of the primary coder was validated against the coding of the second human coder using a kappa score on an overlapping sample of 200 tweets. Series of 200 tweets were double classified until kappa scores greater than 0.7 were achieved and this was used to train a new custom machine classifier that was applied to all the relevant tweets. Inaccuracies in machine coding were refined by human coding of key tweets to retrain the algorithm. Again, machine coding was validated using a kappa score on an overlapping sample of 100 tweets.

For peer review only - http://bmjopen.bmj.com/site/about/guidelines.xhtml

Once relevant tweets were separated into positive, negative and neutral, a random sample of 500 tweets was taken from each of the three subgroups using DiscoverText's random sampling tool. These 1500 tweets were analysed and single coded to assess the predominant themes. Prior to assessment we reviewed previous media arguments for and against MUP,[10] and various public surveys [25] to establish the range of anticipated themes (here we identified 4 positive themes and 8 negative themes). The subsequent process of single coding to assess the predominant themes was an iterative process and when a theme was not congruent with the anticipated themes, it was considered a newly emerging theme and this was added. 2 new themes emerged through this process, 1 positive and 1 negative.

New themes, in addition to those already identified, emerged only in the initial stages of analysis and no new themes emerged in the later stages of analysis (the final 150 tweets of each 500 tweet sample). Thus it was determined that sufficient saturation had been reached, and no additional tweets needed to be examined. The popularity of each theme was also assessed in each random sample.

These three random samples of 500 tweets were also analysed to determine the source of the tweets. A single human coder examined each author's Twitter page. For each tweet/retweet, the username, full name, associated biography and the associated results from an internet search engine (https://www.google.com) were examined to determine the user's background. The same process was used to determine if the source self identified as Scottish or lived in Scotland or not (only 1.6% of Twitter users have their geolocation activated and so inferences must be made from their profile).[26] For example, Twitter profiles contain a space for a user to write their location and if this was a Scottish place it was assumed that the user was Scottish. Some users did not write a location but had explicit references to the place they lived in their tweets. Chi-squared tests were used to determine if any differences in proportions reached statistical significance (p-value <0.05) in categorical variables.

Patient and Public Involvement (PPI)

There was no PPI in the design or conduct of this study. As participants did not explicitly consent to their Tweets being used in this specific research paper, paraphrased examples of Tweets were used to retain anonymity.

Results

74,639 tweets were collected over 14 days. 62,879 of these tweets were manually coded as either 'relevant' to MUP or 'not relevant' by the same coder (LAW). The Naive Bayes algorithm subsequently coded the remaining 11,760 tweets. 500 tweets were coded by both the primary coder and a second coder (AB). Of these 500 tweets, there was a 97% agreement with a kappa score of 0.95. This indicates an excellent level of agreement. In order to validate the coding of the algorithm, 100 tweets were coded by both the primary coder (LAW) and the algorithm. For these 100 tweets there was a 97% agreement with a kappa score of 0.94, providing further reassurance about reliability on relevance.

53,574 (72%) tweets were classified as relevant, while 21,065 (28%) were classified as 'not relevant'. The irrelevant tweets made no reference to the MUP of alcohol in any context. These 53,574 relevant tweets were subsequently classified according to sentiment. 57,801 tweets were manually coded. 18,741 were coded as positive (35%), 14,866 as negative (28%), 17,302 as neutral (32%), and 2665 as not relevant (5%). In the 200 tweets coded by both the primary and secondary coder there was a kappa score of 0.75. The kappa scores were: positive - 0.79, negative - 0.74, neutral - 0.76, not relevant - 0.73. This shows good agreement for sentiment tweets. For 100 tweets coded by both the primary coder (LAW) and the algorithm there was a 96% agreement with a kappa score of 0.94.

From each sentiment (positive, negative and neutral) 500 randomly selected tweets were analysed for predominant themes. These were elaborated through the process of thematically coding each tweet and new themes were added as they occurred until saturation was reached. Twitter based thematic analysis is difficult to automate using machine algorithms due to abbreviations, emoticons and sarcasm[27] and we relied exclusively on human coding. The findings are presented in Table 1. Perceived ability to reduce health harms was the most prominent theme in the positive tweets, skepticism about effects on problem drinkers was the most prominent in the negative tweets, and factual information were the most prominent theme in the neutral tweets. In each of these sentiment categories a small proportion of tweets (ranging from 1.6-5.2%) were

For peer review only - http://bmjopen.bmj.com/site/about/guidelines.xhtml

found to be misclassified. Some were irrelevant but falsely classified as relevant, while some were of another sentiment. It is impossible to say whether it was human or machine coding which produced this error.

Table 1: Results of thematic analysis of positive, negative and neutral tweets with paraphrased examples

Theme of positive tweets	*n*	*%*	*Paraphrased example*
Reduces health harms	352	70.4	Minimum Unit Pricing will decrease hospital admissions and save lives #mupsaveslives
Reduces social harms	13	2.6	This will greatly reduce alcohol-fuelled violence and other countries must follow
Effectively targets the cheapest, strongest alcohol	36	7.2	Strong cider sold at pocket money prices is hugely damaging
Scotland has an alcohol problem and something must be done	26	5.2	This country has an awful relationship with drink - let's try MUP
MUP is an evidence-based policy	5	1.0	The evidence backs MUP, which has been approved by the courts and will be extensively evaluated with a sunset clause
Nil reason given	60	12.0	Excellent work from the SNP!
Incorrectly classified as positive	8	1.6	
Total	500	100	
Theme of negative tweets	*n*	*%*	*Paraphrased example*
Alcoholics will not decrease their alcohol intake	138	27.6	Alcoholics will not buy less but their children will go without so they can get it
Increase in illicit alcohol production and/or encourage cross-border trading	71	14.2	Hoards will rush over the border to stock up on frosty jacks - who would've thought we'd have a booze cruise in 2018
Libertarian	54	10.8	First the sugar tax and now this - the nanny state won't stop
A tax on the poor	52	10.4	Another example of a classist poor-bashing policy
Increase in drug use and/or petty crime	23	4.6	Neds will rob grannies for booze money and the jakeys will turn to drugs instead
Punishes responsible drinkers	17	3.4	A few people can't drink responsibly and now everyone else has to pay the price?
Increases retailer profits	6	1.2	All this will do is line the pockets of billionaires - the supermarkets can't believe their luck
Harms businesses	2	0.4	How many jobs will be lost from this?
Alcohol consumption is a cultural problem	3	0.6	Other countries with cheap alcohol don't have the same problems - the problem isn't to do with the price
Nil reason given	108	21.6	This new alcohol law is embarrassing bs #SNPfail
Incorrectly classified as negative	26	5.2	
Total	500	100	
Theme of neutral tweets	*n*	*%*	*Paraphrased example*
Factual	301	60	Scotland introduces new alcohol law

For peer review only - http://bmjopen.bmj.com/site/about/guidelines.xhtml

Humour	102	20	Great that Scotland are adopting the alcohol pricing design they have trialled for so long at the Edinburgh fringe
Balanced/Unclear Sentiment	82	16	On the one hand it could reduce overconsumption of alcohol, but on the other it could encourage a black market
Incorrectly classified as neutral	15	3	

The random samples of 500 tweets divided by sentiment were next classified according to the background of the Twitter user who posted the tweet. In the case of retweets this was the original tweeter. It was not possible to determine who retweets were made by. The users were divided up into the groups as in Table 2. Miscellaneous users were those accounts who did not fall into any of the other groups and largely consisted of private companies and spam accounts.

Table 2: Source analysis of tweets and retweets from positive, negative and neutral sub-groups

		Original source of tweet or retweet							
		Member of public	*Health/alcohol policy organisation/individual*	*Media/news organisation/individual*	*Alcohol industry-related organisation/individual*	*Celebrity/ public figure*	*Miscellaneous*	*Incorrectly classified as positive/negative/neutral*	*Total*
Positive	*n*	85	275	91	1	17	25	6	500
	%	17	55	18.2	0.2	3.4	5.0	1.2	100
Negative	*n*	287	53	56	5	2	81	16	500
	%	57.4	10.6	11.2	1.0	0.4	16.2	3.2	100
Neutral	*n*	189	45	109	18	10	120	9	500
	%	37.8	9.0	21.8	3.6	2.0	24	1.8	100

For peer review only

Table 3 presents data on source of the tweets, with more positive sentiment demonstrated among those which were likely Scottish. To further examine these data, we used a chi-square test and found that there was a significant difference in sentiment between likely Scottish and not obviously Scottish Twitter accounts (chi-square statistic 22.659, df = 2, p-value <0.001).

For peer review only - http://bmjopen.bmj.com/site/about/guidelines.xhtml

Table 3: Source nationality analysis of tweets and retweets from positive, negative and neutral subgroups						
		Source nationality				
		Likely Scottish	*Not obviously Scottish*	*Unable to view profile*	*Not correctly assigned the right sentiment*	*Total*
Positive	*N*	293	187	14	6	500
	%	58.6	37.4	2.8	1.2	100
Negative	*N*	218	204	65	13	500
	%	43.6	40.8	13	2.6	100
Neutral	*N*	219	259	13	9	500
	%	43.8	51.8	2.6	1.8	100

Discussion

Study findings demonstrate that public opinion on the introduction of MUP in Scotland was somewhat divided, with a slightly higher proportion of positive posts (35%) than negative (28%) or neutral (32%). This was the case particularly in Scotland. These findings mirror previous survey data that suggest a growing proportion of the British public favour MUP than are against it.[11]

Public opinion alone does not dictate alcohol policy and there is often significant industry and political will to resist change. There do, however, remain complex interactions between public opinion and shifts in alcohol policy. Österberg and colleagues[28] demonstrated that a decrease in alcohol excise duty in Finland in 2004 and a subsequent rise in alcohol related harm led to an increase in support for alcohol policies to counteract these trends. In Ireland high levels of alcohol consumption and a doubling of alcohol related street violence over seven years led to public discussions which culminated in increased alcohol taxation, via increased support for alcohol policies.[29] There is some suggestion in this study and the literature on which it draws that Scotland has followed a similar pattern to Finland and Ireland where it appears that an increase in alcohol harms has prompted public discussion putting alcohol policy change on policy agendas. A more nuanced historical study would be needed to investigate how far this is true, and the roles of political actors in relation to public opinion.[30,31]

In 1984 John Kingdon proposed that shifts in public policy require the overlapping of three different factors - the public acknowledgement of a problem, a clear solution to a problem, and also the political will to address the issue. [32] In relation to MUP there was first a public discussion of the harms of alcohol consumption. Researchers and public health experts subsequently paid more attention to restrictive alcohol policies as a solution to this problem, and then the SNP showed the political will to address these alcohol harms [30,31]. These three factors may have overlapped to create a unique 'window of opportunity' to introduce MUP.

Only one other study has examined social media responses (analysing 3061 tweets) to alcohol policy-related developments.[20] The present study is thus the largest conducted on alcohol policy with analysis of 53,574 relevant tweets and the first to use a mixture of human and machine classification. Stautz et al.[20] showed a predominantly negative reaction to updated alcohol guidelines (27.4% negative vs 6.8% positive).

There are several interpretations of the large difference in sentiment between the updated alcohol guidelines and the introduction of MUP. While it is possible that public support for MUP is far greater, it is also

For peer review only - http://bmjopen.bmj.com/site/about/guidelines.xhtml

possible that because Stautz et al.[20] only followed one hashtag (while we followed 29 different synonyms accounting for different terminology and spellings), this procedure yielded a less representative sample. In our study the most popular hashtag relating to MUP was only used in 3.8% of relevant tweets and so we would not recommend searching based on hashtags alone when conducting future alcohol policy-related research.

Thematic analysis of positive tweets showed less variation in arguments supporting MUP than against it. 70.4% of positive tweets focused on the health benefits of MUP and a minority focused on other views. This reflects the introduction of MUP for primarily public health reasons. Furthermore, health/alcohol policy organisations/individuals tweets or retweets were the original sources of the majority of positive tweets surrounding MUP. This suggested a coordinated response by public health organisations focusing on a single message - that MUP reduces alcohol-related health problems. These findings suggest implications for advocacy groups investing in social media to influence public opinion.

Additionally, our study found that 24.9% of the 1500 randomly selected sentiment tweets were made by health/alcohol policy-related Twitter accounts, while Stautz et al.[20] demonstrated 12.4% of tweets relating to the updated UK alcohol guidelines were made by health related individuals/organisations. The responses of health advocacy groups to MUP appears to be more effective than that to alcohol guidelines, in part because of the ongoing failure to implement UK public information campaigns on the new guidelines, and the remarkable refusal of alcohol producers to carry the revised guidelines on alcohol packaging. As Pechey et al.[15] recommend, Scottish policy-makers have given prominence to the expected outcomes of MUP.

Many of the negative themes expressed were similar to alcohol industry framings of the issues from earlier on in the public debate.[9,33] Following on from the industry's attempts to obstruct the implementation of MUP through legal processes, the alcohol actors we identified through Twitter continued to propagate the negative framing of MUP in an attempt to marginalise those arguments based on peer-reviewed literature. Yet, by the time of implementation of the policy, it is striking how little such activity there was by industry actors. It seems more likely that alcohol industry actors pursued other avenues to alter public perception post-MUP implementation rather than that they were inactive, and these were not captured in this study.

The similarity we have demonstrated in findings between Twitter-based research to gauge public perceptions and general population surveys may provide some support for social media-based methods as adjuncts to survey based opinion polling. As set out by our research questions, we showed that 35% of tweets were positive, 28% were negative and 32% were neutral. Similarly the 2011 YouGov survey suggested that 47% supported MUP, while 44% opposed it and 9% were unsure.[12] Likewise the 2015 British Social Attitudes survey suggests that 52% of British adults support MUP, while 25% are against it and 22% are unsure.[11] Gauging public opinion via social media has numerous practical advantages over polling, though validation methods remain to be developed. There will probably be lower costs given that the data are already in the public domain, and machine algorithms can be used to code items with high inter-rater reliability with human coders. Social media research does, however, bring with it new ethical challenges that must be considered by future researchers.[34]

Using the Discovertext software, we were unable to distinguish between original tweets and retweets. It is likely that a significant proportion of the tweets were retweets, but we are unable to gauge what proportion, and this remains a limitation of our study. While retweets are perceived by many as an expression of agreement with the original tweet, this is not always the case. On occasion, retweets were accompanied by a comment from the user. In these circumstances the sentiment of the extra comment was analysed primarily, rather than the sentiment of the retweet.

Other limitations of our work include uncertainty about the inferences about public opinion that can be made from this data. Twitter users are unlikely to be representative of the general population, as they are more likely to be urban dwelling, male, and have higher educational achievement.[35,36] Twitter users tend to hold more extreme views[37] and surround themselves with those who hold similar opinions (in what is known as echo-chambers).[38] This makes any inferences in relation to previous polling data questionable. Furthermore, classification was not perfect and 3.3% of tweets were included in the wrong sentiment group in our random sample of 1500 tweets. It is also possible our results were subject to confounding, for example, by political affiliation. Many accounts provided limited biographical information and so this was

For peer review only - http://bmjopen.bmj.com/site/about/guidelines.xhtml

not measured or adjusted for. In addition, while we demonstrated a high proportion of positive posts, this may not necessarily translate into behaviour change, or speak directly to the possible success of the policy. Nonetheless, it is possible to appreciate the divided nature of public opinion on the introduction of MUP, the nature of the sentiment around it, and key actors involved, and it will be possible to later study how this picture changes when the policy becomes more established.

Ethical Approval
The University of York Health Sciences Research Governance Committee was consulted and recommended that the study did not require ethical approval as the Twitter data used were already in the public domain.

Contributors
LAW, SG and JM were responsible for the original study design. LAW was responsible for primary data coding, analysis and for initial drafting of this report. AB was the second coder. LAW, SG, AB and JM were responsible for subsequent interpretation, editing and rewriting for the report.

Data Availability
Data may be obtained from Discovertext (an online text analytics software - https://discovertext.com) but are not publicly available. Discovertext was used to collect, archive and machine code tweets.

Funding
This work was supported by a Wellcome Trust Investigator Award in Humanities and Social Science (200321/Z/15/Z) to JM.

Declaration of Interests
We declare no competing interests.

References
1. Babor T. Caetano R. 2010. Alcohol: No Ordinary Commodity. 2nd edition. New York: Oxford University Press. ISBN: 0199551146.

2. Monteiro M. 2011. The Road to a World Health Organization Global Strategy for Reducing the Harmful Use of Alcohol. Alcohol Res Health. 34(2). PMID: 22330226.

3. World Health Organization. 2010. Global Strategy to Reduce Harmful Use of Alcohol [Online]. Available at: http://www.who.int/substance_abuse/activities/gsrhua/en/. Accessed 16/08/2018 (Archived by WebCite® at http://www.webcitation.org/71mmbuaiB).

4. Sharma A. Sinha K. Vandenberg, B. 2017. Pricing as a means of controlling alcohol consumption. Br Med Bull. 123(1). PMID: 28910991.

5. Angus C. Holmes J. Pryce R. Meier P. Brennan A. 2016. Model-based appraisal of the comparative impact of Minimum Unit Pricing and taxation policies in Scotland. An adaptation of the Sheffield Alcohol Policy Model version 3 [Online]. Available at: https://www.sheffield.ac.uk/polopoly_fs/1.565373!/file/Scotland_report_2016.pdf. Accessed: 06/05/18 (Archived by WebCite® at http://www.webcitation.org/71mlo3iju).

6. Leon D. McCambridge J. (2006). Liver cirrhosis mortality rates in Britain from 1950 to 2002: an analysis of routine data. Lancet. 367(9504). PMID: 16399153.

7. Scottish Government. 2008. Changing Scotland's relationship with alcohol: a discussion paper on our strategic approach [Online]. Available at: http://www.gov.scot/Resource/Doc/227785/0061677.pdf. Accessed: 06/05/18 (Archived by WebCite® at http://www.webcitation.org/71mmCjRMw).

For peer review only - http://bmjopen.bmj.com/site/about/guidelines.xhtml

8. Meier P. Brennan A. Angus C. Holmes J. 2017. Minimum unit pricing for alcohol clears final legal hurdle in Scotland. BMJ. 359. PMID: 29162561.

9. McCambridge J. Hawkins B. Holden C. 2013. Industry use of evidence to influence alcohol policy: a case study of submissions to the 2008 Scottish government consultation. PLoS Med. 10(4). PMID: 23630458.

10. Hilton S. Wood K. Patterson C. Katikireddi, S. 2014. Implications for alcohol minimum unit pricing advocacy: What can we learn for public health from UK newsprint coverage of key claim-makers in the policy debate? Soc Sci Med. 102(100). PMID: 24565153.

11. NatCen Social Research. 2015. Attitudes to alcohol. Findings from the 2015 British Social Attitudes survey [Online]. Available at: http://bsa.natcen.ac.uk/media/39126/bsa-attitudes-to-alcohol-final.pdf. Accessed: 09/05/2018 (Archived by WebCite® at http://www.webcitation.org/71mm05C1f).

12. YouGov. 2011. 'Pocket money' prices [Online]. Available at: https://yougov.co.uk/news/2011/01/25/pocket-money-prices-alcohol/. Accessed: 07/05/2018 (Archived by WebCite® at http://www.webcitation.org/71mmQgn15).

13. Hagger M. Lonsdale A. Baggott R. Penny G. Bowen M. 2011. The Cost of Alcohol: The Advocacy for a Minimum Price per Unit in the UK [Online]. Available at: http://alcoholresearchuk.org/downloads/finalReports/FinalReport_0082. Accessed 07/05/18 (Archived by WebCite® at http://www.webcitation.org/71mltCAb8).

14. Somerville C. Marteau T. Kinmonth A. Cohn S. 2015. Public attitudes towards pricing policies to change health-related behaviours: a UK focus group study. Eur J Public Health. 25(6). PMID: 25983329.

15. Pechey R. Burge P. Mentzakis E. Suhrcke M. Marteau M. 2014. Public acceptability of population-level interventions to reduce alcohol consumption: a discrete choice experiment. Soc Sci Med. 113. PMID: 24858928.

16. Erskine S. Maheswaran R. Pearson T. Gleeson D. 2010. Socioeconomic deprivation, urban-rural location and alcohol-related mortality in England and Wales. BMC Public Health. 10(99). PMID: 20184763.

17. Giesbrecht N. Lalomiteanu A. Anglin L. Adlaf E. 2007. Alcohol marketing and retailing: Public opinion and recent policy developments in Canada. Journal of Substance Use. 6. doi:10.1080/14659890701262189.

18. Giesbrecht N. and Livingston M. 2014. Public perceptions and alcohol policies: Six case studies that examine trends and interactions. Drug Alcohol Rev. 33(3). doi:10.1111/dar.12139.

19. Statista. 2018. Number of monthly active Twitter users worldwide from 1st quarter 2010 to 4th quarter 2017 (in millions) [Online]. Available at: https://www.statista.com/statistics/282087/number-of-monthly-active-twitter-users/. Accessed: 08/05/2018 (Archived by WebCite® at http://www.webcitation.org/71mmJR0Bd).

20. Stautz K. Bignardi G. Hollands G. Marteau T. et al. 2017. Reactions on Twitter to updated alcohol guidelines in the UK: a content analysis. BMJ Open 7(2). doi:10.1136/bmjopen-2016-015493.

21. Thompson L. Rivara F. Whitehill J. 2013. Prevalence of Marijuana-Related Traffic on Twitter, 2012–2013: A Content Analysis. Cyberpsychol Behav Soc Netw. 18(6). PMID: 26075917.

22. Cole-Lewis H. Pugatch J. Sanders A. Varghese A. Posada S. Yun, C. Schwarz M. Auguston E. 2015. Social Listening: A Content Analysis of E-Cigarette Discussions on Twitter. J Med Internet Res. 17(10). PMID: 26508089.

For peer review only - http://bmjopen.bmj.com/site/about/guidelines.xhtml

23. Huang J. Kornfield R. Szczypka G. Emery S. 2014. A cross-sectional examination of marketing of electronic cigarettes on Twitter. Tob Control. 23(3). PMID: 24935894.

24. Rivers C. Lewis B. 2014. Ethical research standards in a world of big data. F1000Research. 38(1-10). doi:10.12688/f1000research.3-38.v1.

25. Alcohol Policy UK. 2016. British Social Attitudes survey 2015: is support for minimum pricing growing? [Online]. Available at: http://www.alcoholpolicy.net/2016/09/social-survey-attitudes-2015-is-support-for-minimum-pricing-growing.html. Accessed 04/05/18 (Archived by WebCite® at http://www.webcitation.org/71mlXdIwf).

26. Leetaru K. Wang S. Cao G. Padmanabhan A. Shook E. 2013. Mapping the global Twitter heartbeat: The geography of Twitter. First Monday. 18(5). doi:10.5210/fm.v18i5.4366.

27. Thelwall M. Buckley K. Paltoglou G. 2012. Sentiment strength detection for the social Web. Journal of the American Society for Information Science and Technology. 63(1). doi:10.1002/asi.21662.

28. Österberg E. Lindeman M. Karlsson T. 2014. Changes in alcohol policies and public opinions in Finland 2003-2013. Drug Alcohol Rev. 33(3). PMID: 24628708.

29. Hope A. 2014. The ebb and flow of attitudes and policies on alcohol in Ireland 2002–2010. Drug Alcohol Rev. 33(3). PMID: 24628739.

30. Holden C. Hawkins B. 2014. 'Whisky gloss': The alcohol industry, devolution and policy communities in Scotland. Public Policy & Administration. 28. doi:10.1177/0952076712452290.

31. McCambridge J. Hawkins B. Holden C. 2014. Vested interests in addiction research and policy. The challenge corporate lobbying poses to reducing society's alcohol problems: insights from UK evidence on minimum unit pricing. Addiction. 109(2). PMID: 24261642.

32. Béland, D. Howlett, M. 2016. The Role and Impact of the Multiple-Streams Approach in Comparative Policy Analysis. Journal of Comparative Policy Analysis. 18(3).

33. Hawkins B. McCambridge J. 2014. Industry Actors, Think Tanks, and Alcohol Policy in the United Kingdom. Am J Public Health. 104(8). PMID: 24922137.

34. Golder S. Ahmed S. Norman G. Booth A. 2017. Attitudes Toward the Ethics of Research Using Social Media: A Systematic Review. J Med Internet Res. 19(6). PMID: 28588006.

35. Mellon J. Prosser C. 2017. Twitter and Facebook are not representative of the general population: Political attitudes and demographics of British social media users. Research and Politics. 1(9). doi:10.1177/2053168017720008.

36. Mislove A. Lehmann S. Ahn Y. Onella J. Rosenquist J. 2011. Understanding the demographics of Twitter users. Proceedings of the Fifth International AAAI Conference on Weblogs and Social Media. AAAI Press. ISBN: 9781577355052.

37. Barberá P. Rivero G. 2014. Understanding the Political Representativeness of Twitter Users. Social Science Computer Review. 33(6). doi:10.1177/0894439314558836.

38. Garimella K. De Francisci Morales G. Gionis A. Mathioudakis M. 2018. Political Discourse on Social Media: Echo Chambers, Gatekeepers, and the Price of Bipartisanship. WWW 2018: The 2018 Web Conference. doi:10.1145/3178876.3186139.

For peer review only - http://bmjopen.bmj.com/site/about/guidelines.xhtml
